# TGF-β1 Promotes Zika Virus Infection in Immortalized Human First-Trimester Trophoblasts via the Smad Pathway

**DOI:** 10.3390/cells11193026

**Published:** 2022-09-27

**Authors:** Quang Duy Trinh, Ngan Thi Kim Pham, Kazuhide Takada, Chika Takano, Shihoko Komine-Aizawa, Satoshi Hayakawa

**Affiliations:** Department of Pathology and Microbiology, Division of Microbiology, School of Medicine, Nihon University, Tokyo 173-8610, Japan

**Keywords:** Zika, trophoblasts, pregnancy, infection, congenital Zika syndrome, first trimester, TGF-β1, transforming growth factor-beta 1, Tyro3, AXL

## Abstract

The Zika virus (ZIKV) is well known for causing congenital Zika syndrome if the infection occurs during pregnancy; however, the mechanism by which the virus infects and crosses the placenta barrier has not been completely understood. In pregnancy, TGF-β1 is abundant at the maternal–fetal interface. TGF-β1 has been reported to enhance rubella virus binding and infection in human lung epithelial cells. Therefore, in this study, we investigate the role of TGF-β1 in ZIKV infection in the immortalized human first-trimester trophoblasts, i.e., Swan.71. The cells were treated with TGF-β1 (10 ng/mL) for two days before being inoculated with the virus (American strain PRVABC59) at a multiplicity of infection of five. The results showed an enhancement of ZIKV infection, as demonstrated by the immunofluorescent assay and flow cytometry analysis. Such enhanced infection effects were abolished using SB431542 or SB525334, inhibitors of the TGF-β/Smad signaling pathway. An approximately 2-fold increase in the virus binding to the studied trophoblasts was found. In the presence of the Smad inhibitors, virus replication was significantly suppressed. An enhancement in Tyro3 and AXL (receptors for ZIKV) expression induced by TGF-β1 was also noted. The results suggest that TGF-β1 promotes the virus infection via the Smad pathway. Further studies should be carried out to clarify the underlying mechanisms of these findings.

## 1. Introduction

Zika virus (ZIKV) belongs to the genus *Flavivirus* and the family Flaviviridae. Although this virus is generally a causative agent of a mild illness with fever, rash, and joint pain [1,2], it has been known worldwide for causing congenital Zika syndrome (CZS) in children born to mothers with the ZIKV infection during pregnancy [3,4]. Other complications include miscarriage, stillbirth, and intrauterine growth restriction if the infection occurs in early pregnancy [5]. The above unfavorable pregnancy outcomes with ZIKV infection result in severe medical and public health consequences [6,7,8,9]. Since the spread of ZIKV in the Americas, and the potential link with microcephaly in babies of pregnant women who were infected, scientists and authorities in health policymakers worldwide have paid more attention to research into this virus; in particular, vaccine development against ZIKV has been vigorously practiced [10,11,12,13,14,15,16].

Although ZIKV is well known for the severe consequences caused by its infection during pregnancy, the mechanisms of transplacental infection have not been well understood. For viral entry, some molecules such as TAM (Tyro3, AXL, Mer) have been known as receptors for ZIKV in some cell types or tissues [17,18]. However, the roles of these proteins in ZIKV infections have not been well studied in the maternal–fetal interface. In addition, possible factors affecting the virus infection at this interface have not been investigated.

It is known that the risk of a structural birth defect among infants born to mothers with ZIKV infection during pregnancy ranges from 5 to 10%, with higher incidences when the infection occurs in the first trimester [19,20]. The first trimester of a healthy pregnancy is featured by a balance between the invasion of trophoblast cells and fetal–maternal immune tolerance, with the predominant role of regulatory T cells (Treg cells) being indispensably regulated by TGF-β1 [21,22,23]. TGF-β1 plays essential roles in cell growth and differentiation, trophoblast cell invasion, maintenance of fetal–maternal immune tolerance, and uterine spiral artery remodeling [24,25,26]. TGF-β1 is abundant at the maternal–fetal interface. Immune cells, such as Treg cells, and non-immune cells, particularly first-trimester trophoblast cells, secrete TGF-β1 [24,27,28,29,30]. Elevated concentrations of TGF-β1 in maternal plasma and placenta were noted in preeclamptic pregnancies [31,32]. TGF-β1 has been reported to enhance rubella virus binding and infection in human lung epithelial cells [33]. However, its roles in ZIKV infection in early pregnancy have not been reported.

In this study, we investigate the possible roles of TGF-β1 in ZIKV infection in the first trimester of pregnancy using an in vitro model. By performing the virus infection in an immortalized human first-trimester trophoblast cell line, Swan.71, we found that TGF-β1 promotes ZIKV binding and infection in these trophoblasts via the Smad pathway.

## 2. Materials and Methods

### 2.1. Cell Culture

The Swan.71 cells used in this study were kindly provided by Dr. Gil Mor (Wayne State University, Detroit, MI, USA). These cells were derived from the telomerase-mediated transformation of a 7-week cytotrophoblast isolate described by Straszewski-Chavez [34]. The cells were cultured in RPMI 1640 medium (Gibco-Invitrogen, Tokyo, Japan) supplemented with 10% fetal bovine serum (FBS), 10 mM HEPES (Invitrogen), 0.1 mM nonessential amino acids (Invitrogen), and 1 mM sodium pyruvate (Invitrogen) 100 units/mL penicillin-streptomycin (complete medium). Vero cells were purchased from the Japanese Collection of Research Bioresources Cell Bank and cultured in Dulbecco’s modified Eagle’s medium (DMEM) (Gibco-Invitrogen, Tokyo, Japan) supplemented with 10% FBS and 100 units/mL penicillin-streptomycin. All cells were cultured in monolayers at 37 °C in a humidified 5% CO_2_ incubator.

### 2.2. Zika Virus

The ZIKV strain (American strain PRVABC59) was transferred from Nagasaki University (Nagasaki, Japan) under an agreement with the Centers for Disease Control and Prevention (Atlanta, GA, USA). The viral stock solution was prepared by propagating the virus in Vero cells. Viral titers were estimated with the TCID50 method or flow cytometry (FCM) analysis.

### 2.3. Cell Treatment with TGF-β1

Swan.71 cells were seeded in 6-well plates (1 × 10^5^ cells/well), 24-well plates (2.5 × 10^4^ cells/well), and 96-well plates (5 × 10^3^ cells/well) in a complete medium. The following day, the cells were treated with TGF-β1 (10 ng/mL), with or without its Smad pathway inhibitors, SB431542 (10 μM) or SB525334 (10 μM), for 2 days before the viral infection experiments or collection of cellular RNAs and lysates for real-time PCR and Western Blot experiments, respectively. In other experiments, the cells were infected with ZIKV without any pretreatments. However, at 4 hpi, TGF-β1 or the inhibitor (same concentrations as described earlier) was added into the culture to investigate their roles in the virus replication by performing the progeny viral quantification in the supernatants collected at 48 hpi.

### 2.4. Cell Viability Assay

Trophoblast cells were cultured in a 96-well plate and subjected to TGF-β1 and its inhibitor treatments described above. Cell viability was measured using a Cell-Counting Kit-8 (Dojindo Laboratories, Kumamoto, Japan) according to the manufacturer’s instructions. The cell density in each well was measured at 450 nm using a microplate reader (iMark Microplate Absorbance Reader, Bio-Rad, Hercules, CA, USA).

### 2.5. Western Blotting

Cells grown in 6-well plates were subjected to the TGF-β1 treatments described above. The supernatant was removed 48 h posttreatment, and the cells were washed and lysed in 70 μL of cell lysis buffer (Cell Signaling Technology, Danvers, MA, USA). The protein concentrations in the lysates were quantified using a DC Protein Assay (Bio-Rad Laboratories, Inc., Hercules, CA, USA). Cell lysates were loaded onto a NuPAGE 4–12% Bis-Tris protein gel (Invitrogen) and separated by electrophoresis. The separated proteins were transferred to polyvinylidene fluoride membranes (Invitrogen), and nonspecific binding sites were blocked using 1% BSA in phosphate-buffered saline (PBS) containing 0.05% Tween 20. Membranes were incubated with a primary rabbit monoclonal anti-vimentin antibody (ab92547, Abcam, Cambridge, UK; 1:3000) or a rabbit α-Tubulin antibody (2144S, Cell Signaling Technology; 1:3000) at 4 °C overnight. Membranes were incubated with a horseradish peroxidase-conjugated secondary antibody (Cell Signaling Technology) for 30 min at room temperature (RT) and visualized with a luminescent image analyzer (Image Reader LAS-4000 Mini, Fujifilm, Tokyo, Japan).

### 2.6. Viral Infection

For in vitro viral infection, after treatment, the cells were washed with a serum-free medium and then incubated with the virus at multiplicities of infection (MOI) of 5 for a total of 1 h in a 37 °C, humidified 5% CO_2_ incubator with gentle shaking every 5~10 min. The supernatant was removed, the cells were washed, and the medium was replaced with a fresh medium containing 2% FBS. Percentages of the ZIKV- infected cells at 24 h post-infection (hpi) were determined by FCM analysis, and the ZIKV-infected cells at 48 hpi were observed using an immunofluorescence assay. Negative control cells (mock-infected, infected with heat-inactivated ZIKV, or not exposed to the primary antibody during the staining procedures) and positive controls (ZIKV-infected Vero cells) were performed in parallel for comparison.

The infectivity of the collected supernatants was determined using the TCID50 method or FCM analysis. For the TCID50 method, briefly, 1 day before infection, Vero cells were seeded onto 96-well plates (10^4^ cells/well). One hundred microliters of serially 10-fold diluted supernatants or virus stocks were used to infect the Vero cells in quadruplicate. A total of 50 μL of fresh medium containing 2% FBS was added on day 5, and the results were collected on day 9 pi after fixation and staining with crystal violet. The TCID50 was calculated using the Reed–Muench method.

For viral titration by FCM analysis, a 30 μL volume of serial 3-fold dilutions of the supernatants was used to infect (in duplicate) freshly seeded Vero cells in a 96-well plate (4 × 10^4^ cells/well). Medium containing 2% FBS and NH_4_Cl was added at 6 hpi to prevent a second round of infection (final concentration of NH_4_Cl, 20 mM). The cells were collected at 24 hpi and subjected to intracellular staining of the ZIKV capsid protein. The viral titer (in infectious units (IUs)) of a sample was calculated as the average of 3 titers measured in 3 consecutive wells with a percentage of ZIKV-infected cells lower than 40% and higher than 0.3%, as described previously [35].

### 2.7. Immunofluorescence (IF) Assay

As described above, the cells cultured on glass coverslips in 6-well plates were subjected to the ZIKV infection. Separate negative controls subjected to mock infection, heat-inactivated ZIKV inoculation, and staining with normal rabbit serum were established. The supernatant was removed at 48 hpi, and the cells were fixed with cold methanol for 5 min, washed with PBS, and incubated with the rabbit polyclonal anti-ZIKV capsid antibody (GTX133317, GeneTex, Hsinchu, Taiwan) for 1 h at RT. The cells were washed with PBS and incubated with an Alexa 488-conjugated goat anti-rabbit IgG (H + L) secondary antibody (ab150081, Abcam) solution for 30 min at RT. The samples were counterstained with 4′,6-diamidino-2-phenylindole dihydrochloride (DAPI) (Lonza, Walkersville, MD, USA). After washing, the coverslips were mounted with VECTASHIELD Mounting Medium (Vector Labs, Burlingame, CA, USA), and fluorescence images were acquired using a fluorescence microscope (FLoid Cell Imaging Station; Life Technologies, CA, USA).

For the cell-surface staining of Tyro3 or AXL, after the supernatant was removed, the cells were fixed with 4% paraformaldehyde (PFA). After blocking, the cells were incubated with one of a primary antibody, a rabbit polyclonal anti-Tyro3 antibody (Proteintech, Rosemont, IL, USA), or a rabbit polyclonal anti-AXL antibody (GTX129407, GeneTex), followed by the previously mentioned second antibody (ab150081, Abcam).

### 2.8. FCM Analysis

The studied trophoblast cells were collected at 24 hpi using the detachment medium (RPMI containing 2.9 mM EDTA, 2% FBS, Live/Dead Staining Solution (Live/Dead Fixable Near-IR Dead Cell Stain Kit, Thermo Fisher Scientific, Waltham, MA, USA). In brief, the cells were incubated for approximately 1 h after adding the detachment medium [36]. After suspension and centrifugation, the cells were washed with a staining buffer (STB, cold PBS containing 5% goat serum and 2 mM EDTA) then fixed with PFA and permeabilized using a BD Cytofix/Cytoperm Fixation/Permeabilization Solution Kit (BD Biosciences, San Diego, CA, USA). Intracellular staining was performed with a rabbit polyclonal anti-ZIKV capsid antibody (GTX133317, GeneTex) for 30 min at RT. The cells were washed and incubated with a goat anti-rabbit IgG H&L (Alexa Fluor^®^ 488) secondary antibody (ab150081, Abcam) solution for 30 min at RT. The cells were then subjected to FCM analysis after washing and fixation. For each sample, at least 5000 gated events were collected and analyzed on a BD FACSVerse cytometer using BD FACSuite software (version 1.2; BD Biosciences, San Diego, CA, USA). Separate negative controls were also established without viral inoculation or incubated with heat-inactivated ZIKV.

To investigate the expression of Tyro3 and AXL, the cells were stained and collected using a previously described two-step protocol for preparing adherent cells [36]. Briefly, after culture and treatments, the supernatant was removed. The detachment medium containing appropriate primary antibodies, i.e., a rabbit polyclonal anti-Tyro3 antibody (28513-1-AP, Proteintech) or a rabbit polyclonal anti-AXL antibody (GTX129407, GeneTex), was added and incubated in an incubator for approximately 1 h. After one wash with STB, the cells were incubated with a goat anti-rabbit IgG H&L (Alexa Fluor 488) secondary antibody (ab150081, Abcam). The cells were washed, fixed with PFA, and subjected to FCM analysis.

### 2.9. Virus Binding Assay

The cells were seeded onto 96-well plates and then subjected to the TGF-β1 and the inhibitor treatments described above. The cells were washed once with ice-cold PBS, then a viral binding buffer (0.1% BSA in PBS containing 0.1% sodium azide) was added for incubation on ice for 10 min. The cells were inoculated with ZIKV on ice for 1 h with gentle shaking every 5 min. The cells were gently washed 3 times with ice-cold PBS, subjected to cell-surface staining for ZIKV (on ice, 1 h for each staining step), collected using STB buffer containing 2.9 mM EDTA, and then fixed with PFA. The ZIKV-positive cells were determined using FCM analysis. For double staining of the cells for ZIKV and Tyro3 (or AXL), the first antibodies used were a rabbit polyclonal anti-Tyro3 (or anti-AXL) antibody and a mouse anti-ZIKV envelope protein antibody (GTX634155, GeneTex). The combined second antibodies used were a goat anti-rabbit IgG H&L (Alexa Fluor 488) and a goat anti-mouse (Alexa Fluor 647).

### 2.10. RNA Extraction and RT-qPCR

The Swan.71 cells were cultured in 24-well plates and subjected to TGF-β1 treatment with or without TGF-β1 inhibitors as described above. The total mRNA was extracted using TRIzol reagent (Life Technologies, Tokyo, Japan), and any contaminated genomic DNA was removed by treatment with DNAse I (TaKaRa Bio, Inc., Otsu, Japan). Real-time RT-PCR was performed using a One-Step TB Green Prime-Script PLUS RT-PCR Kit (Perfect Real Time) (TaKaRa Bio) in a QuantStudio5 Real-Time PCR System (Applied Biosystems, MA, USA). The following primer sequences were used: Vimentin, sense, 5′-GAC GGT TGA AAC TAG AGA TGG AC-3′ and antisense, 5′-CTT GCG CTC CTG AAA AAC TGC-3′, as well as peptidylprolyl isomerase A (PPIA), sense, 5′-ATG CTG GAC CCA ACA CAA AT-3′ and antisense, 5′-TCT TTC ACT TTG CCA AAC ACC-3′. The results were analyzed using the delta–delta Ct method.

To quantify the ZIKV progeny produced during the post-infection period, the viral RNA genome was extracted from the supernatants using the QIAamp Viral RNA mini kit (Qiagen, Hilden, Germany) according to the manufacturer’s instructions. The viral RNA was then subjected to real-time PCR procedures. The viral RNA standard was prepared by the MEGAscript T7 Transcription Kit (Thermo Fisher Scientific) using T7 promoter primers as the followings: sense, 5′-TAA TAC GAC TCA CTA TAG CCT TGG ATT CTT GAA CGA GGA-3′, antisense, 5′-TTT TTT TTT TTT TTT AGA GCT TCA TTC TCC AGA TCA A-3′; ZIKV primers, sense, 5′- CCT TGG ATT CTT GAA CGA GGA -3′; antisense, and 5′- AGA GCT TCA TTC TCC AGA TCA A -3′.

### 2.11. Statistical Analysis

Analysis of variance was used to analyze the results. A *p*-value < 0.05 obtained using the Tukey–Kramer test and Statcel 4 software (OMS Publishing, Inc., Tokorozawa, Saitama, Japan) was significant. Data are presented as the mean ± SEM.

## 3. Results

### 3.1. Expression of Tyro3 and AXL on the Swan.71 Cells and Their Upregulation upon the TGF-β1 Treatment

**Figure 1:**
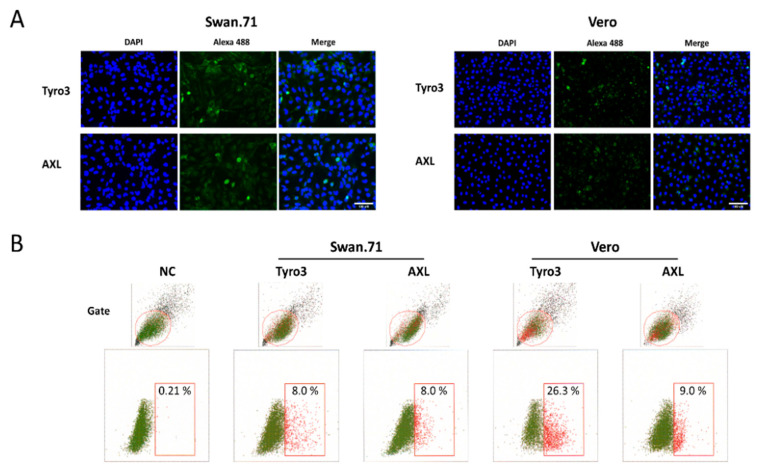
Expression of Tyro3 and AXL on the surface Swan.71 cells. (**A**) Immunofluorescence assay. The cells were cultured on glass coverslips in 6-well plates and stained for Tyro3 or AXL. Scale bar: 100 μM. (**B**) Flow cytometry analysis. Trophoblast cells were cultured in a 96-well plate and stained for Tyro3 or AXL. Numbers displayed inside each panel correspond to the percentage of positive cells for Tyro3 or AXL of the parent-gated population. (**A**,**B**) Data obtained from Vero cells performed in parallel were used as comparative references.

The first-trimester trophoblast cells, Swan.71, differentiated into mesenchymal cells with strong vimentin expression while showing very low cytokeratin and hardly detectable E-cadherin, as mentioned in our previous studies [37,38]. These cells express Tyro3 and AXL, which are putative cellular receptors for ZIKV (Figure 1A). However, less than 10% (5% to 8%) of the gated cells positive for Tyro3 or AXL were frequently noted by FCM analysis (Figure 1B). 

No significant changes in the cell viability upon the treatment of TGF-β1 and/or its Smad pathway inhibitors (SB431542, SB525334) were noted (Figure S1A). Although no apparent change in the protein-level expression was observed in the Western Blotting analysis for vimentin expression, its mRNA expression increased significantly at 24 h post-treatment of TGF-β1 compared to the mock-treated group (the cells cultured in a serum-free medium (SF) without the added exogenous TGF-β1). In the presence of the Smad pathway inhibitors, its mRNA expression was decreased to a level lower than that of the SF group (Figure S1A). The cell-surface expression of Tyro3 and AXL increased significantly under TGF-β1 treatment, as noted by FCM analysis, and it returned close to the normal level in the presence of a Smad pathway inhibitor (Figure S2).

### 3.2. Low Susceptibility Zika Virus in Swan.71 Cells and Its Enhancement in the Presence of TGF-β1

**Figure 2:**
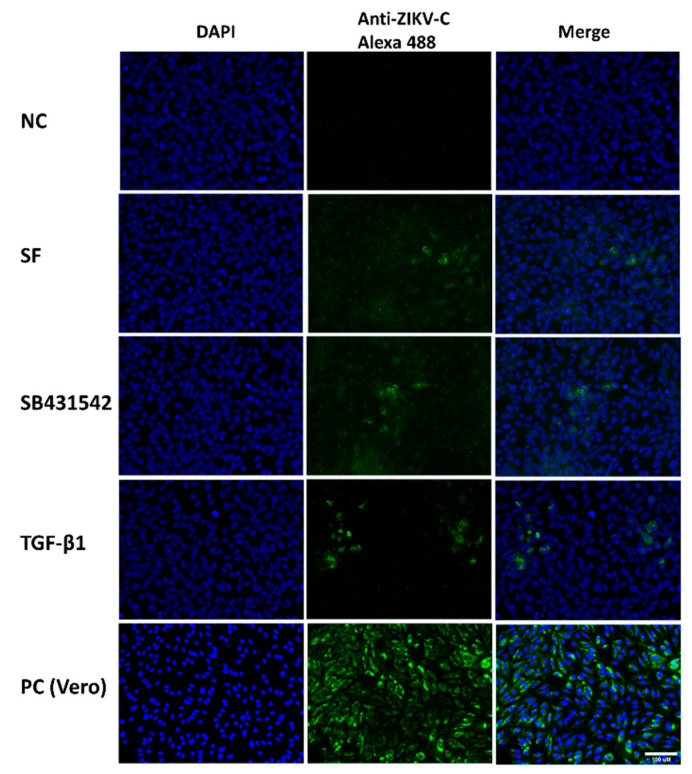
Representative microscopy images of Swan.71 cells infected with ZIKV at 48 hpi. The cells were fixed and labeled with a rabbit polyclonal anti-ZIKV viral capsid antibody followed by an Alexa 488-conjugated goat anti-rabbit IgG (H + L) secondary antibody (green). Nuclei were stained with DAPI (blue). ZIKV-infected Vero cells were used as positive controls (PC). Trophoblast cells that were mock infected, incubated with heat-inactivated ZIKV, or stained with rabbit serum were used as negative controls (NC). Images are representative of 3 independent experiments. SF, Swan.71 cells cultured in the serum-free medium; SB431542, an inhibitor of TGF-β1/Smad pathway; ZIKV-C, Zika virus capsid. Scale bar: 100 μM.

**Figure 3:**
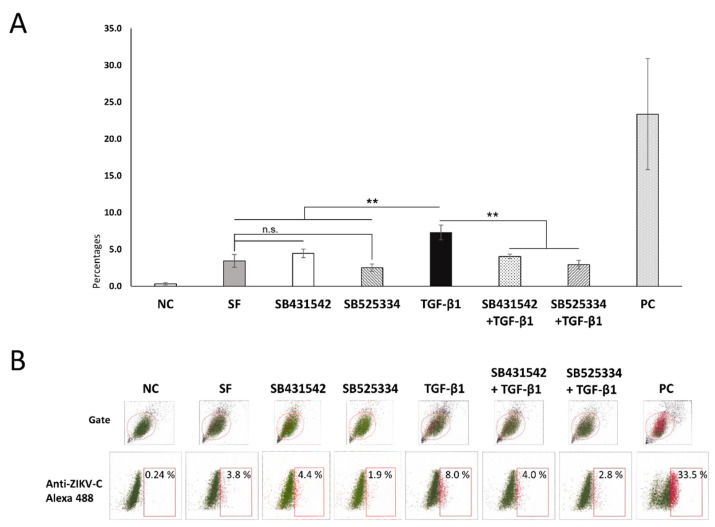
Percentages of the studied trophoblast Swan.71 cells positive for ZIKV as determined by FCM analysis at 24 hpi. (**A**) Mock-infected cells using a culture medium were used as negative controls (NC). Vero cells infected with ZIKV performed in parallel were used as positive controls (PC). The results are expressed as the mean of at least triplicate experiments in each group, and the graph is representative of three independent experiments. n.s., not significant; **, *p* < 0.01. Representative images of FCM analysis. (**B**) Numbers displayed inside each panel correspond to the percentage of the cells positive for ZIKV capsid protein of the parent-gated population.

**Figure 4:**
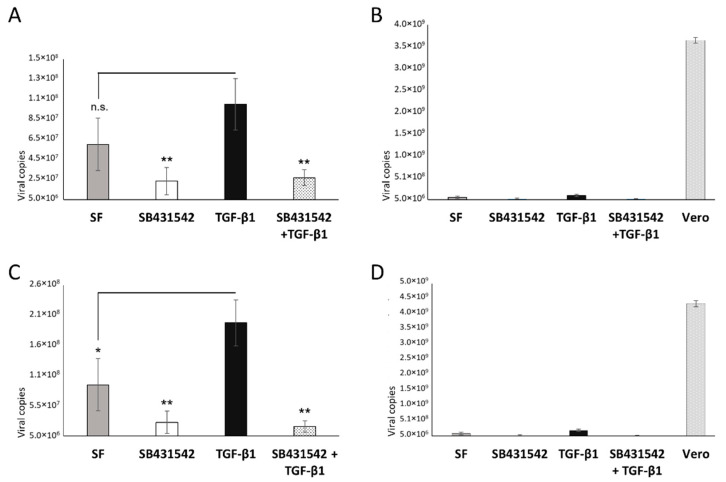
Quantifying viral progeny production in the supernatants collected at 48 hpi by real-time PCR. (**A**,**B**) Pre-infection treatments of TGF-β1 and/or its inhibitor SB431542. Swan.71 cells were cultured and pre-treated with TGF-β1 and the inhibitor before ZIKV infection. (**C**,**D**) Post-infection treatments of TGF-β1 and/or its inhibitor SB431542. The Swan.71 cells were infected with ZIKV without any pretreatments. TGF-β1 and/or the inhibitor were added to the culture at 4 hpi. Supernatant collected from ZIKV-infected Vero cells was used as the positive control. The results are expressed as the mean of at least triplicate experiments in each group, and each graph is representative of three independent experiments. n.s., not significant; *, *p* < 0.05; **, *p* < 0.01. (**B**,**D**) Data obtained from Vero cells were included in the graphs for comparative references.

For the viral infection results, the IF images at 48 hpi showed scattered signals of infection of ZIKV in the trophoblast cells, much lower in comparison to the positive control, Vero cells. This observation was supported by the FCM analysis, showing an average of 2–3% of the trophoblast cells infected with ZIKV at 24 hpi and no increase during the post-infection period. In contrast, more than 20% of the Vero cells were infected with this virus strain at 24 hpi and reached over 40% at 48 hpi. After adding the exogenous TGF-β1, the IF assay noted an enhancement of the intracellular localization of the ZIKV capsid protein (Figure 2). Subsequently, an average 2-fold increase in the ZIKV infection rate of these trophoblast cells was confirmed by FCM analysis, reaching approximately 8% of the studied Swan.71 cells. In the presence of TGF-β1/Smad pathway inhibitors, SB431542 or SB525334, either alone or along with the exogenous TGF-β1, the percentages of the ZIKV-infected trophoblast cells decreased close to the SF group (Figure 3). Compared to the Vero cells, the amounts of progeny virus released into supernatants were much lower for the studied Swan.71 cells (Figure 4).

A statistically significant increase in the virus-progeny production was not noted for the cells pre-treated with the TGF-β1 compared with the SF group by real-time PCR (Figure 4A,B). However, for the cells infected with ZIKV without any pretreatments, a significant enhancement in viral replication was noted for the ZIKV-infected cells cultured in the presence of exogenous TGF-β1 in the post-infection period (Figure 4C,D). In addition, its constant decrease in viral replication was observed in the presence of the well-known TGF-β1/Smad pathway inhibitor SB431542 (Figure 4A,C).

### 3.3. Enhancement in ZIKV Binding to the TGF-β1-Treated Cells via the Smad Pathway

**Figure 5:**
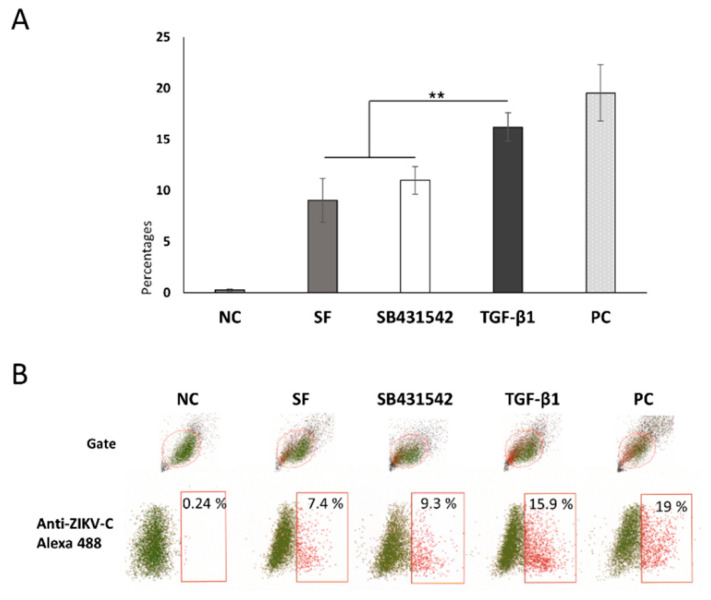
Enhancement of Zika virus binding to the trophoblast cells upon TGF-β1 treatment via the Smad pathway. (**A**) Percentages of the ZIKV-positive cells in the virus binding assay determined by FCM analysis. The results are expressed as the mean (±SEM) of at least triplicate experiments in each group, and the graph is representative of three independent experiments. **, *p* < 0.01. (**B**) Representative images of flow cytometry analysis. Numbers displayed inside each panel correspond to the percentage of the cells positive for ZIKV capsid protein of the parent-gated population. The results are expressed as the mean (±SEM) of at least triplicate experiments in each group, and the graph is representative of two independent experiments. **, *p* < 0.01.

The virus binding assay showed an average of 7 to 8% of the studied Swan.71 cells positive for ZIKV by FCM analysis. Under the TGF-β1 treatment, an approximately 2-fold increase in the ZIKV-positive rates was noted. In the presence of SB431542, it returned close to that found in the SF group (Figure 5).

The double staining of ZIKV and Tyro3 or AXL in the virus binding assay showed no exclusive association of any of the two proteins with ZIKV-infected cells. In each case, the majority of the ZIKV-infected cells was negative for the targeted protein (Figure S3).

## 4. Discussion

Although ZIKV was discovered in the middle of the last century, this virus that causes mild disease had not been paid attention until the link of its infection in pregnant women with the outcome of congenital Zika syndrome in their children [1,2,3,4,5]. In recent years, basic research into this virus has been conducted widely, along with worldwide researchers’ efforts in ZIKV vaccine development [13,14,15,16]. Investigations into the mechanisms of the transplacental infection of ZIKV and the CZS have been conducted on large clinical scales, in vitro and in animal models [3,39,40]. Immortalized human first-trimester trophoblast cells have been used in in vitro settings [41]. In this study, by using a high MOI to assure every single cell has a chance to come in contact with at least one virus particle theoretically, we found that this ZIKV strain has a low susceptibility in this first-trimester immortalized trophoblast cell line.

The first trimester of pregnancy is a critical period for major fetal organogenesis. Congenital syndromes derived from perinatal viral infections often occur in this crucial period [19,42]. Regarding the mechanism for protecting the fetus against possible infections, trophoblast cells are considered the first placental barrier. The first-trimester trophoblast cells have been known as being resistant or having low susceptibility against various viruses in vitro [37,43]. It is worth noting that the findings of resistance or low susceptibility of trophoblast cells in artificial culture may not reflect the actual condition of a given intrauterine viral infection during the first trimester. As congenital syndromes resulting from some viral infections often occur in the first trimester, it is suggested that there may exist unknown factors that favor the intrauterine viral infection of these trophoblast cells. In our recent study, we found that the low susceptibility of the rubella virus in the first-trimester trophoblast cells was enhanced drastically under glucose stress conditions [44].

In line with the above hypothesis trend, in this study, we investigated the possible roles of TGF-β1, an essential growth hormone in early pregnancy and abundant at the maternal–fetal interface, in ZIKV infection in first-trimester trophoblasts. We found that the low susceptibility of ZIKV in the studied trophoblast cells was increased in the presence of an exogenous TGF-β1, causing the infection rate to double in percentages. This finding was supported by a double increase in the virus binding to the TGF-β1-treated trophoblast cells. As these trophoblast cells can secrete TGF-β1, the results suggest that the TGF-β1, including its endogenous source, may be one factor that favors the ZIKV infection of trophoblasts in early pregnancy.

In this study, as described in the Results section, a statistically significant difference in the virus progeny production was not noted when comparing the TGF-β1-treated and the mock-treated cells, the cells cultured in a serum-free (SF) medium without the added exogenous TGF-β1. This unclear change might be due to the existing endogenous TGF-β1 in the SF group. This explanation is supported by a significantly constant decrease in viral-progeny production under the SB431542 treatment, inhibiting both exogenous and endogenous TGF-β1. For the cells infected with ZIKV without any pretreatments, a significant enhancement in viral replication was observed in the presence of exogenous TGF-β1 during the post-infection period. This finding confirms the role of TGF-β1 in ZIKV replication.

It was noted that the percentages of ZIKV-positive cells in a virus binding assay were higher than those of the cells collected at 24 hpi. One possible main reason is that the cell density might increase significantly after 24 h of culture. Another possibility is that the low susceptibility of the ZIKV in the newly formed Swan.71 cells might limit their infection, and the virus-susceptible cell contact might not be effectively induced. In addition, every ZIKV-binding cell might not turn into an infected cell due to the suppression effect on viral replication of interferon-beta secreted from these cells [45].

Regarding virus entry, Tyro3, AXL, and Mer (TAM) have often been suggested as cellular receptors for ZIKV; of these, AXL was suggested as a critical factor [17,18]. However, recent studies reported that the functions of TAM in the context of viral entry might vary depending on cell types or experimental models, suggesting the existence of multiple viral-entry mechanisms [39,40,46,47,48,49]. In addition, none of the previously suggested receptors were validated in the transplacental infection setting.

In this study, we observed a low expression for both Tyro3 and AXL on the studied trophoblast cell surface, and their expression was upregulated under the TGF-β1 treatment. In a previous study, the low expression for Tyro3 was observed in the human placenta but not with AXL [50]. In the virus binding assay with double staining of the ZIKV and each of the Tyro3 and AXL, the results of no exclusive association of any of these two proteins with ZIKV-infected cells and the majority of the ZIKV-infected cells negative for the targeted protein in each case imply that the ZIKV entry process in these trophoblast cells may involve different pathways. This observation agrees with previous studies conducted in mice or using the third-trimester trophoblast cells JEG-3 [46,51]. However, further studies using appropriate approaches such as silent gene methods should be considered as clarifying the above suggestion.

In summary, this study showed a low susceptibility of first-trimester trophoblast cells using the immortalized human trophoblast cell line Swan.71. The study also found a role of TGF-β1 in promoting Zika virus binding and replication in these trophoblast cells via the Smad pathway. Our study has the natural limitation of an in vitro study. Therefore, these findings must be interpreted appropriately in clinical settings. Further studies should be considered using placenta explant or other trophoblast cells in an intrauterine-mimicking culture condition.

## 5. Conclusions

In conclusion, this study reports the low susceptibility of ZIKV in an immortalized human first-trimester trophoblast cell line, Swan.71. In addition, the study suggests that TGF-β1, an essential growth hormone for cell development and differentiation in early pregnancy, promotes ZIKV infection in these trophoblast cells via the Smad pathway. The findings provide more insight into the mechanisms of ZIKV infection in the first trimester of pregnancy.

## Data Availability

The raw data supporting the conclusions of this article can be made available by the authors without undue reservation.

## Supplementary Materials

The following supporting information can be downloaded at: https://www.mdpi.com/article/10.3390/cells11193026/s1, Figure S1. Cell viability assay of the studied trophoblast Swan.71 cells cultured in the presence of TGF-β1 and/or its Smad pathway inhibitors; Figure S2. Upregulation of cell surface expression of Tyro3 and AXL of the trophoblast Swan.71 cells upon the TGF-β1 treatment via the Smad pathway; Figure S3. Representative images of FCM analysis of the virus binding assay with double staining of ZIKV and Tyro3 or AXL.
