# Supplementary material for: TGF-β1 Promotes Zika Virus Infection in Immortalized Human First-Trimester Trophoblasts via the Smad Pathway
Source: Cells. 2022 Sep 27;11(19):3026. doi: 10.3390/cells11193026 (PMC9562857; doi:10.3390/cells11193026)
Supplement: Supplementary file 1 [file cells-11-03026-s001.zip › cells-1865304-supplementary.pdf]

Supplementary Figures

A

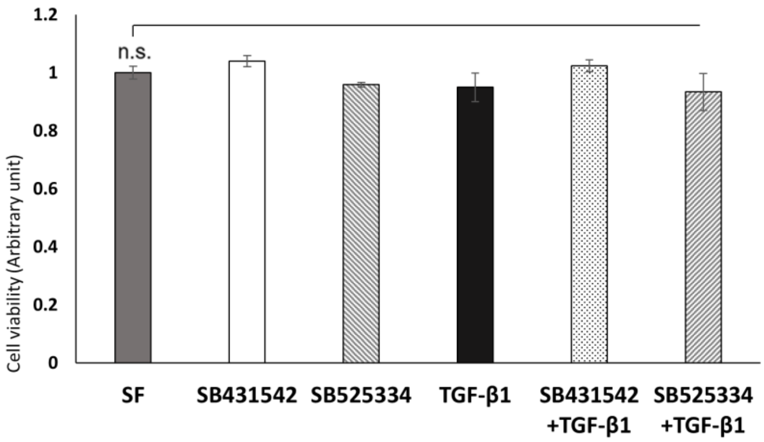

B

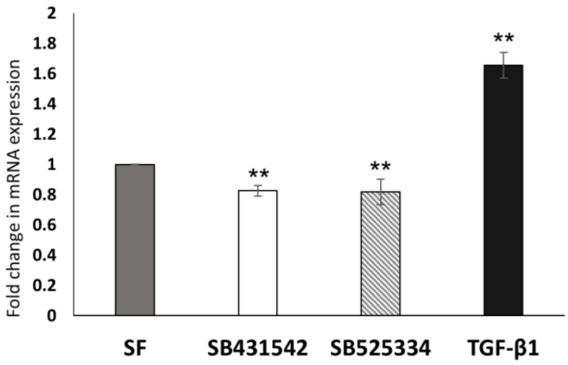

Figure S1. A. Cell viability assay of the studied trophoblast Swan.71 cells cultured in the presence of TGF-β1 and/ or its Smad pathway inhibitors. The obtained absorbance values were normalized to those of the cells cultured in the SF medium. **B. Changes in mRNA expression of vimentin under TGF-β1- or its inhibitor treatments at 24 h.** Data shown are the means of fold-change of relative expression values (normalized to the internal control PPIA gene expression) ± SEM. (**A**) and (**B**): the results are expressed as the mean of at least triplicate experiments in each group, and each graph is representative of three independent experiments. Abbreviation: n.s., not significant. **, $p < 0.01$.

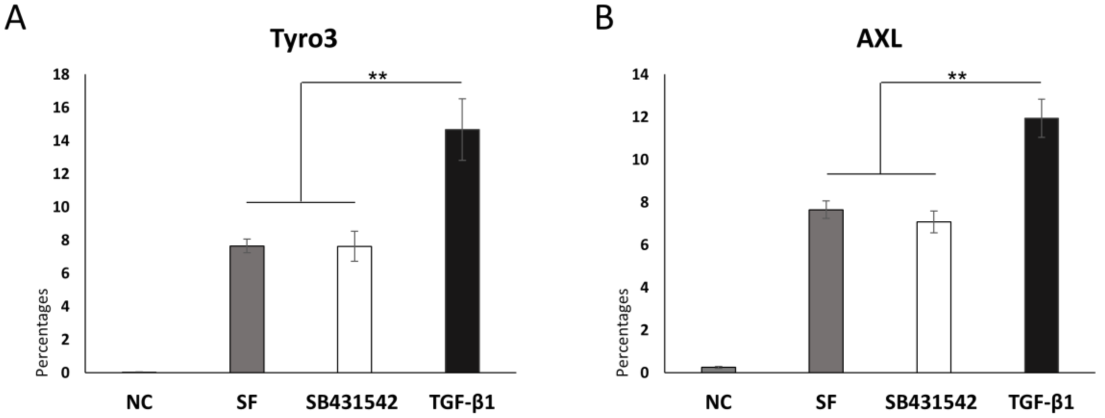

Figure S2. Upregulation of cell surface expression of Tyro3 (A) and AXL (B) of the trophoblast Swan.71 cells upon the TGF-β1 treatment via the Smad pathway. After being treated with TGF-β1 or its Smad pathway inhibitor SB431542, the cells were stained for Tyro3 or AXL using a previously described two-step protocol for preparing adherent cells, and then analyzed by FCM. Data are shown as percentages of the cells positive for Tyro3 or AXL in the parent-gated populations; and are expressed as the mean (± SEM) of at least triplicate experiments in each group, and each graph is representative of three independent experiments. **, $p < 0.01$.

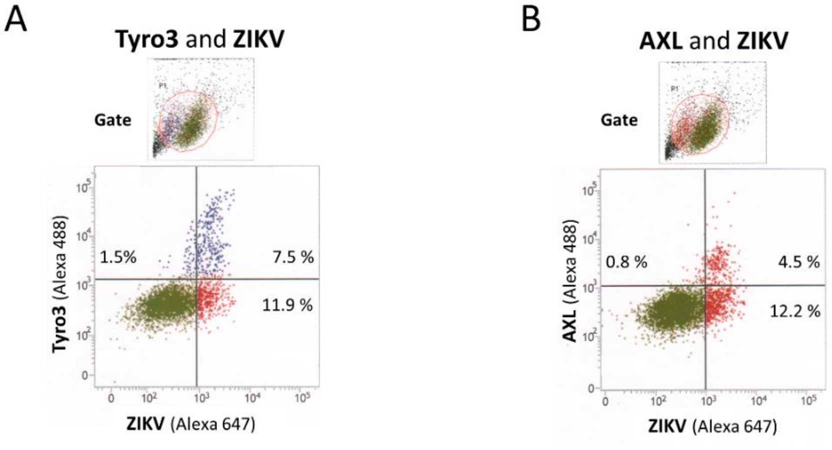

Figure S3. Representative images of FCM analysis of the virus binding assay with double staining of ZIKV and Tyro3 or AXL. The second antibodies used were a goat anti-rabbit IgG H&L (Alexa Fluor 488) for Tyro 3 and AXL, and an Alexa 647- conjugated goat anti-mouse IgG (H + L) secondary antibody for ZIKV. Data are shown as percentages of the target cells in the parent-gated populations.
